# Decreased dengue transmission in migrant worker populations in Singapore attributable to SARS-CoV-2 quarantine measures

**DOI:** 10.1093/jtm/taaa228

**Published:** 2020-12-03

**Authors:** Jue Tao Lim, Borame Lee Dickens, Janet Ong, Joel Aik, Vernon J Lee, Alex R Cook, Lee Ching Ng

**Affiliations:** Saw Swee Hock School of Public Health, National University of Singapore and National University Health System, Singapore; Saw Swee Hock School of Public Health, National University of Singapore and National University Health System, Singapore; Environmental Health Institute, National Environmental Agency, Singapore; Environmental Health Institute, National Environmental Agency, Singapore; Saw Swee Hock School of Public Health, National University of Singapore and National University Health System, Singapore; Ministry of Health, Singapore; Saw Swee Hock School of Public Health, National University of Singapore and National University Health System, Singapore; Environmental Health Institute, National Environmental Agency, Singapore

**Keywords:** COVID-19, non-pharmaceutical interventions, Asia, arboviruses, social distancing

## Abstract

**Background:**

We examined the impact of SARS-CoV-2 social distancing and quarantine policies on dengue transmission in the general and migrant worker populations in Singapore.

**Methods:**

We utilized all nationally reported dengue cases in the general and migrant worker populations from 1 January 2013 to 31 May 2020. A difference-in-difference identification strategy was used to determine the effects of social distancing and quarantine policies on reported dengue case counts over time, whilst controlling for weather patterns, seasonality, age and population size.

**Results:**

A reduction of 4.8 dengue cases per age band among migrant workers was attributable to quarantine policies, corresponding to a total reduction of around 432 reported dengue cases over 10 weeks. In the general working population, an increase of 14.5 dengue cases per age band was observed, which corresponds to a total increase of around 1450 reported dengue cases in the same time period. There is an expected relative risk reduction in dengue transmission for the migrant worker population at 0.635 due to quarantine policy and a relative risk increase for the general working population due to social distancing policies at 0.685.

**Conclusions:**

Migrant workers experienced a reduced risk of dengue when they were confined to their dormitories as part of the COVID-19 social distancing measures. Our study highlights the vulnerability of migrant workers under normal working conditions.

## Background

The ongoing coronavirus disease 2019 (COVID-19) pandemic, caused by the Severe Acute Respiratory Syndrome Coronavirus 2 (SARS-CoV-2), has led to the widespread implementation of non-pharmaceutical interventions to curb transmission in the general population and subpopulations, including migrant workers.^1^ COVID-19 infection risk in migrant workers living in dormitories can be elevated due to their dense living environment, inability to socially isolate and access to healthcare and prevention.^2^^,^^3^

Singapore has a foreign workforce of ~1.4 million, of whom ~300 000 work in construction, cleaning or related industries.^4^ A majority resides in dormitories—high-density housing with shared living spaces.^5^ In the 6 months between March and August 2020, over 50 000 PCR confirmed SARS-CoV-2 cases were reported in migrant worker dormitories, versus ~2000 in the general population of >5 million, highlighting the differential risks in COVID-19 transmission among the two groups.^3^^,^^4^^,^^6^^,^^7^

Dengue is a global problem which increasingly affects travellers, including migrant workers who pursue work outside their home country.^8^ In tandem with the COVID-19 pandemic, Singapore experienced a record-breaking dengue outbreak in 2020, with an unprecedented 30 000 diagnosed cases reported at the end of September. A 2-month imposition of social distancing measures nationally in response to the COVID-19 outbreak, similar to lockdown in other countries, contributed to the rise in dengue cases in the general population,^9^^,^^10^ presumably due to increased peridomestic contact between humans and mosquitoes. In the same period, strict quarantine policies have been enacted in migrant worker dormitories due to the large number of cases in these locales and to prevent onward community spread of COVID-19.^3^ It is not clear, however, how social distancing and quarantine measures imposed on foreign workers might have altered their risk of acquiring dengue. Construction sites, where a large proportion of migrant workers reside in the work day, are typically hard to manage environments, being previously demonstrated to be nexuses for both dengue and Zika transmission.^11^^,^^12^ Answering this question may contribute to our understanding of the risks associated with workplace exposures, and the impacts of the shift of exposure risk from workplaces to homes, for both the general community and migrant worker populations.

This study aimed to identify the potential causal effects of confining migrant workers to dormitory sites on reported dengue cases by considering social distancing and quarantine policies as a natural experiment.

## Methods

The notification of all confirmed cases of dengue in Singapore is legally mandated by the Ministry of Health for the purpose of disease surveillance and control. We obtained reported case counts of dengue in Singapore by 5-year age bands from 2013 to 2020 (data were available to epidemiological week [e-week] 23, 2020) and analysed the data on a weekly timescale for the community and migrant workers.

We controlled for the effects of weather on potential vector breeding, using weather data obtained from Meteorological Services Singapore.^13^ Maximum and mean temperature, and relative humidity, were included as these were correlated with mosquito population activity in Singapore.^14^

Two months of enhanced social distancing measures lasted from 7 April (e-week 15) to 1 June (e-week 23).^4^ During this period, around 95% of workplaces were closed, including all schools and recreational facilities, and all construction sites.^15^ To prevent widespread transmission of COVID-19 in the dormitories, all migrant construction workers were quarantined in their residences during this period. This therefore changed the ratio of time exposed to mosquitoes at home versus at (mostly) air-conditioned workplaces—for the general population—or (mostly) worksites—for migrant construction workers, and thereby the risk of dengue infection if these locations have heterogeneous risk.

The treatment effects of social distancing and quarantine policies on dengue exposure in the general community were therefore expected to be experienced more strongly by working-age adults and school-going children (i.e. those aged 5–65), and also migrant workers living in dormitories. Those outside these age ranges were assigned as the control group. These individuals were likely to have limited change in their mobility patterns from social distancing policies, as <50% of individuals below 5 attend childcare centres for full-day programmes and <1% of retired individuals reside in elderly care facilities.^16^^,^^17^ The majority of these individuals spent their time in residences or very localized childcare facilities within the neighbourhood both before and during social distancing policy implementation.

A difference-in-difference (DiD) identification strategy was used to determine the causal effects of social distancing and quarantining policy on dengue case counts over the 10-week period. We took observations of reported dengue case counts in three treatment groups separately: (1) working adults aged 20–65 years during lockdown, (2) schooling population aged 5–19 years old during lockdown, (3) migrant workers in dormitories in quarantine. The total control group for each separate comparison comprised of cases in all age bands before social distancing policies, and those in ages 5 or younger and over 65 during social distancing policies. The effects of social distancing policies were then measured as

$$ {y}_{i,t}= X\beta +{\sum}_jI{\left(\mathrm{policy}\right)}_{i,t,j}{\delta}_j+{\epsilon}_{i,t} $$

where ${y}_{i,t}$ denotes the number of reported dengue case counts at time point $t$ for the age band $i$. $I{\big(\mathrm{policy}\big)}_{i,{t}_{,j}}$ is an indicator variable taking value 1 if the social distancing or quarantine policy effect is active at that time point for that treatment group, and is 0 otherwise. The policy effects, ${\delta}_j$, are the primary estimands of interest. The error term ${\epsilon}_{i,t}\sim \mathrm{N}\big(0,{\sigma}^2\big)$ is normally distributed with assumed constant variance ${\sigma}^2$. $X$ is a matrix of controlling variables which include (1) time trend—to control for the temporal dependence of dengue case counts over time, as well as the near-term trends in dengue transmission potential, (2) past epidemiological weeks—to control for seasonality, (3) year fixed effects—to control for year specific risk,^18^ (4) community or migrant worker populations—to control for differential risk within each populations, (5) age groups in 5 year age bands—to control for immunity buildup and age specific dengue risk and (6) maximum, mean temperature and relative humidity of up to 4 weeks lag—to control for thermal forcing and stress on vector population growth.^19^ The coefficient values for these controlling variables are denoted by $\beta$. These confounders were sequentially added to fully ensure robust identification of both social distancing and quarantine policy effect estimates and to ascertain that our estimated policy effects were not due to other phenomena which may potentially lead to changes in dengue case counts before and after policy implementation.

The relative risk reduction $RR{R}_j$ due to the ${j}^{th}$ policy was then recovered by taking the fraction of the estimated number of cases averted/admitted from the policy ${\delta}_j$ and the average difference between the number of cases observed during the policy period for the treated group $\frac{1}{J}\sum{y}_{\mathrm{treated}}$ and the estimated number of cases averted/admitted from the ${j}^{th}$ policy. $J$ denotes the number of observations in the treated group during the time points where the ${j}^{th}$ policy was instituted:

$$ RR{R}_j=\frac{\delta_j}{\frac{1}{J}\sum \left({y}_{\mathrm{treated}}-{\delta}_j\right)} $$

## Results

After controlling for all available potential confounders, we observed a reduction of 4.8 reported dengue cases per five-year age band per week (Table 1, M5, Treatment 3, 95% CI: 1.5 to 8.1), attributable to the quarantine policy for migrant workers in Singapore aged 45 and below, corresponding to a relative risk reduction of 68.5%. In total, there is an estimated reduction of ~432 reported dengue cases over the quarantine period of 10 weeks among all foreign workers.

**Table 1:** Estimates of treatment effects on dengue case counts per 5-year age band due to social distancing (SD) policy

			Treatment effect on:		
	Population	SD	General community		Foreign workers
	Age group		(1) 5–19	(2) 20–69	(3) 20–65
Model	Controlling for:				
M1	Uncontrolled	0.06 (−0.08, 0.20)	9.2^***^ (2.5, 16.0)	29.8^***^ (25.1, 34.4)	−5.36^*^ (−10.1, −0.6)
M2	Population	0.10 (−0.03, 0.23)	6.1 (−0.2, 12.4)	26.6^***^ (22.3, 30.9)	2.7 (−1.7, 7.2)
M3	Population age	−0.09 (−0.20, 0.03)	4.5 (−1.0, 10.1)	17.2^***^ (13.4, 21.0)	−2.1 (−5.9, 1.8)
M4	Population age year & e-week	0.34^***^ (0.24, 0.44)	1.6 (−3.0, 6.2)	14.2^***^ (11.0, 17.5)	−5.0^**^ (−8.3, −1.7)
M5	Population age year & e-week Weather	0.33^***^ (0.22, 0.43)	1.8 (−2.8, 6.4)	14.5^***^ (11.2, 17.7)	−4.8^**^ (−8.1, −1.5)

Quantities are number of cases attributable/prevented per 5-year age band per group per week. Parentheses contain 95% confidence intervals.

Significant at

^***^ *P*<0.001,

^**^ *P*<0.01,

^*^ *P*<0.05.

These findings contrasted with an increase of 14.5 reported dengue cases per 5-year age band in the working age groups of the general population which did not live in dormitories (Table 1, M5, Treatment 2, 95% CI: 11.2 to 17.7) that was attributable to social distancing policies, corresponding to a relative risk increase of around 63.5%. Among the general population, therefore, there was an increase of ~1450 reported dengue cases during the social distancing period. An increase in the number of cases was also found among schooling individuals but this effect had 95% confidence intervals which contained 0 (Table 1, M5, Treatment 1: 1.8, 95% CI: −2.8 to 6.4).

## Conclusions

The implementation of COVID-19 quarantine measures among migrant workers living in dormitories, mostly limited their movements to within managed accommodation and away from their usual place of work, decreased the number of reported dengue cases among that population. This contrasted with the impact in the general community, wherein dengue cases rose during lockdown due to social distancing policies. This dichotomy implied between group differences in mosquito exposure at worksites and residences.

Outdoor construction and similar worksites are known to be problematic for vector breeding control due to the presence of persistent surface water from rainfall or manmade inputs.^11^ In Singapore, although <5% of dengue case clusters were recorded in construction sites between 2013 and 2016, clusters that did form at construction sites were substantially more likely to develop into major clusters.^11^ These construction site clusters have also led to spill-over dengue transmission in surrounding residential areas.^11^ Similar evidence was found in Rio de Janeiro, Brazil, with a preliminary correlation observed between dengue incidence rates and construction rubble accumulation.^20^ Additionally, in Zhanjiang Prefecture, China, a dengue outbreak began at a construction site with 102 cases,^21^ and in Vientiane, Lao People’s Democratic Republic, a construction site was a point source for an outbreak of 27 cases.^22^

Dormitory accommodation is centrally managed, designed for efficiency, and often have communal toilets to facilitate cleaning and prevention of mosquito breeding. The paucity of space for personal effects may also reduce mosquito breeding opportunities. This study suggests that time spent at dormitories leads to lower exposure to dengue-infected mosquitoes than time spent at worksites. In addition, many of these dormitories are sited well away from the residential areas that house the general population, offering increased protection from public and private residential developments that were historically more prone to dengue outbreaks.^9^

In terms of accommodation, migrant construction worker dormitories in Singapore are well-ventilated but have relatively higher population densities compared to public housing, which likely contributes to dengue transmission risk should mosquitoes exhibit multiple host biting behaviour.^23^ There has therefore been a strong drive over the years to improve the housing standards of these dormitories to prevent dengue transmission within these residences, coupled with frequent checks by National Environment Agency Officers for mosquito breeding sites.^24^ The results suggest that transmission risk was consequently reduced at dormitory sites relative to workplaces where despite the ongoing initiatives, workers are at higher risk of dengue transmission in workplaces, relative to their place of residence.^25^

Within the general community, dengue transmission risk increased during the period of intensified social distancing measures, suggesting that home residences remained as key transmission sites.^9^ Domestic and relatively localized outdoor transmission was identified as a primary pathway in Thailand where 60% of dengue cases living <200 m apart were identified as being from the same transmission chain as opposed to 3% which were separated 1 to 5 km apart.^26^ In Brazil, 70.4% (95% CI: 58.2% to 79.8%) of individual transmission events occurred within 500 m,^27^ and in Vietnam, households were described as foci for dengue transmission in highly urbanized areas.^28^ For Singapore, increased dengue transmission during the social distancing intervention period may be partially attributed to an overall higher exposure to mosquito populations in the home relative to workplaces, which may be air conditioned and therefore relatively segregated from outdoor mosquito breeding and resting sites.

Our results illustrate the opposite effect of social distancing measures on dengue risk between the migrant construction worker and community populations during the 2020 dengue and COVID-19 epidemics of Singapore. The current dominance of DENV-3 is problematic due to its lower seroprevalence and consequent population wide immunity,^29^ requiring greater mosquito population surveillance and intense vector management, which is particularly challenging during the social distancing and quarantine intervention period. An increase in dengue cases in the general community may be attributed to longer dwelling times and a higher probability of mosquito bites within the home, with the inverse among migrant construction workers who may be at relatively higher risk of dengue transmission at working sites.^30^

Several limitations are however present in our study. The implementation of social distancing and quarantine policies could result in under reporting of dengue cases as individuals may be less able to seek professional medical treatment. Under-reporting may also result from the additional burden SARS-CoV-2 places on health systems during the height of the pandemic, which may decrease the number of non-COVID-19 patients seeking treatment. This may bias downwards the estimated policy effects on the incidence of dengue. When more data become available, compartmental models could be used to adjust for the reporting rate. The lack of spatially resolved dengue case data also means that the identification strategy can only be conducted at a national scale, which may not be able to account for differences in pre-post policy effects on dengue transmission across both space and time, though these policies considered were implemented on a national scale, so the impact of this should be limited.

The study highlights the vulnerability of migrant workers to dengue infection under their usual working conditions at worksites. Increased measures for migrant worker protection against dengue should be considered. Such measures are likely to benefit the general population who may live within close proximity to construction sites.
